# Dominance of *Zygosaccharomyces* and shifts in bacterial pathways: Effects of antimicrobials on composition and diversity of the *Ceratitis capitata* bacterial and fungal microbiome

**DOI:** 10.1371/journal.pone.0335811

**Published:** 2025-11-12

**Authors:** Maria Cecilia Rasuk, Alfonsina Palladini, Andrea Moyano, Viviana Díaz, Antonella Giudice, Gisela Castillo, Solana Abraham, Juan Rull, Anja Poehlein, Rolf Daniel, Julian Rafael Dib

**Affiliations:** 1 Citrus Biocontrol Lab, Pilot Plant for Microbiological Industrial Processes (PROIMI), National Scientific and Technical Research Council (CONICET), San Miguel de Tucumán, Tucumán, Argentina; 2 Eco-ethological Research Laboratory of Fruit Flies and their Natural Enemies, Biological Pest Control Division, PROIMI-CONICET, Tucumán, Argentina; 3 Genomic and Applied Microbiology & Göttingen Genomics Laboratory, Institute of Microbiology and Genetics, Georg-August University of Göttingen, Göttingen, Germany; 4 Institute of Microbiology, Faculty of Biochemistry, Chemistry and Pharmacy, National University of Tucumán, Tucumán, Argentina; National Institute of Agricultural Research - INRA, MOROCCO

## Abstract

The Mediterranean fruit fly (*Ceratitis capitata* Wied.) is an agricultural pest of significant economic importance. This species has been globally managed using the Sterile Insect Technique (SIT). Insects, including tephritid flies, harbor a diverse gut microbiota that plays critical roles in their physiology, behavior, and overall fitness, suggesting that microbial communities may profoundly influence the biology of this pest. The aim of this study was to characterize the fungal and bacterial gut microbial communities of *C. capitata* from Tucumán, Argentina, and to assess their response to antimicrobial treatment using amplicon-based 16S rRNA gene and ITS region sequencing. Both control and treated flies were dominated by Proteobacteria (bacteria) and *Zygosaccharomyces* (fungi). Antimicrobial treatment induced significant shifts in bacterial and fungal composition, reducing diversity and altering gut community structure. Untreated flies exhibited a diverse and structured bacterial gut community dominated by the family Enterobacteriaceae, while antibiotic-treated communities were dominated by Rhizobiaceae. Despite these shifts, fungal communities in both treated and untreated groups were consistently dominated by the genus *Zygosaccharomyces*. Functional predictions revealed notable changes in metabolic pathways following antibiotic treatment, including increased gene abundance for ABC transporters and the phosphotransferase system, and decreased representation of genes involved in antibiotic biosynthesis and two-component systems. These results indicate significant alterations in bacterial metabolism and stress response mechanisms induced by the treatment. Such changes may help explain the underperformance of irradiated, mass-reared males within the context of SIT. This study provides new insights into the structural and functional dynamics of the *C. capitata* gut microbiome under disturbance. These findings have implications for understanding the ecological roles of microbial communities in this pest and their potential impact on fly health and fitness. Identification of dominant gut bacterial and fungal groups may support the development of probiotic diets, enhancing the efficiency of SIT application.

## Introduction

The Mediterranean fruit fly, *Ceratitis capitata*, hereafter referred to as Medfly, a pest of major economic importance, is known for its broad host range and high adaptability. The Medfly, originally from Sub-Saharan Africa, has invaded tropical and subtropical biomes of all continents [1]. Its success as an agricultural pest has been partly attributed to its gut microbiome, which plays key roles in host nutrition, detoxification, immunity, environmental stress resistance, and reproductive fitness [2]. The Medfly is controlled worldwide through the application of the Sterile Insect Technique (SIT), in which large numbers of flies are reared, sterilized, and released into the field to suppress wild populations of the pest [3]. In its native range, SIT application against Medfly is limited to South Africa. Mass rearing and irradiation have been shown to negatively impact sterile male performance, possibly because these processes alter the Medfly gut microbiota [4,5]. Indeed, larval diets in mass rearing facilities, include food preservatives that inhibit fungal and bacterial growth [6]. Recent efforts to improve sterile male performance have focused on developing bacteria-based probiotic diets to restore the microbiome of mass-reared flies and increase their performance under SIT [7], although the role of yeasts in the microbiome—and their potential as probiotics—remains unexplored [8]. Studies on tephritid gut microbiomes have consistently identified Proteobacteria, particularly Enterobacteriaceae, as the dominant bacterial taxa across various geographic regions and host plants [8–11]. These bacteria contribute to essential functions such as nitrogen fixation, sugar metabolism, and pathogen protection, underscoring their ecological and physiological significance. In Medfly, Enterobacteriaceae have been recognized as a core component of the microbiome in multiple populations worldwide [8–10]. However, antibiotic treatments have demonstrated the plasticity of these bacterial communities, with shifts in taxonomic composition and metabolic pathways observed after disturbance [6–12]. In contrast, the fungal communities (mycobiome) within the gut of Medfly remain largely unexplored. Studies on other tephritid species suggest that fungi may play complementary or unique roles in insect attraction and metabolism [13–15]. Emerging evidence indicates that fungi contribute to the production of volatile organic compounds (VOCs), the breakdown of complex carbohydrates, nutrient cycling, and gut homeostasis [16–17]. Yeasts have been recognized as essential symbionts of Tephritidae. In Medfly, they are a source of protein affecting longevity, with low-yeast diets leading to reduced protein accumulation in larvae [18–19], whereas enriched yeast diets promote higher pupal recovery and weight [20]. It has been shown that live yeasts inoculated into insect diets promote greater development than diets with inert yeasts [21], suggesting an active role in nutrient cycling. Moreover, studies in other insects suggest that yeasts may provide critical steroid precursors necessary for development, as observed in *Scaptotrigona depilis*, in which *Zygosaccharomyces* plays a key role during pupation [22–23]. A previous study, conducted in our laboratory, characterized the culturable yeast communities in the gut of Medfly from Tucumán, Argentina, identifying *Zygosaccharomyces* as the sole dominant genus [24]. The synergistic presence of bacterial and fungal communities has been linked to increased resistance to desiccation and elevated temperatures in the Medfly [24], potentially mediated by enhanced cuticular wax production [25]. These findings underscore the significance of fungal communities in the Medfly microbiome and highlight the need for further investigation using culture-independent approaches to unravel microbial diversity and dynamics.

In this study, we aim to characterize the bacterial and fungal communities of Medfly from Tucumán, Argentina, with a particular focus on how antimicrobial treatment influences microbiome diversity, composition, and functional profiles. We expected that treated flies would possess a less diverse and abundant microbiome, or alternatively, they would experience shifts in microbiome composition. These insights into the microbial ecology of Medfly may result in the development of targeted probiotic diets to enhance the performance of mass-reared irradiated males in SIT programs.

## Methodology

### Experimental design

Medfly infested fallen fruits were collected under bitter orange trees (*Citrus aurantium*) planted as ornamental trees in public parks and public sidewalks in the municipalities of Tucumán and Yerba Buena, Argentina. The larval diet of flies from our experiment was naturally infested host fruit pulp (orange). Fallen fruit from such trees requires no special collection permit. We choose to use wild flies in order to sample the original and complete natural Medfly microbiome. Larvae and pupae were recovered, and the emerged adults were divided into two groups, both provided with diet saccharose (57.9%) (Ledesma S.A., Jujuy, Argentina), hydrolyzed yeast (14.5%) (Yeast Hydrolyzed Enzymatic, MP Biomedicals®), hydrolyzed corn (27.3%) (Gluten Meal, ARCOR®, Tucumán, Argentina), and vitamin E (0.3%) (Parafarm®, Buenos Aires, Argentina) and water *ad libitum*. At three days of age, the water for one group (treated, T) was replaced with a solution containing ciprofloxacin (5 µg/ml), piperacillin (100 µg/ml), and methylparaben (0.1%), following a previously described protocol [24]. Our design did not include a control to separate the effect of antibiotics and antifungals. Our goal was to expose wild flies to antimicrobial conditions encountered in mass rearing facilities. This design cannot rule out interactions between treated microbial and fungal communities. In this study, we experimentally reduced the microorganisms in the gut of *C. capitata* using a combination of two antibiotics, ciprofloxacin and piperacillin, and a yeast inhibitor, methylparaben. Ciprofloxacin is a broad-spectrum fluoroquinolone antibiotic that directly inhibits DNA synthesis by targeting essential enzymes involved in DNA replication, transcription, and repair. Piperacillin is a beta-lactam antibiotic from the penicillin class that binds to essential proteins required for cell wall construction and maintenance, inhibiting its synthesis, which leads to lysis and death [26]. Both antibiotics are effective against a wide range of gram-negative bacteria, including the Enterobacteriaceae family [27], which are dominant in the Medfly gut [10]. Methylparaben inhibits fungal growth by disrupting protein synthesis and cell membrane permeability, leading to toxic buildup and cell death [28]. The other group (control, C) received tap water. After one week of treatment, males (M) and females (F) from each group were dissected separately under sterile conditions using a stereomicroscope (Arcano ZTX 1065). Each sample consisted of ten extracted guts, homogenized in 1 mL of TE buffer containing 0.2% Tween 80. A total of 12 samples (6 males: control and treated [MC and MT], and 6 females: [FC and FT]) were processed, representing triplicates for each sex and treatment.

### DNA extraction

Prior to DNA extraction, the surface of each insect was sterilized in 70% ethanol and rinsed three times in sterile PBS. Total DNA was extracted from the gut samples using the MasterPure™ Complete DNA and RNA Purification Kit (Epicenter, Wisconsin, USA), following the manufacturer’s instructions.

### Sequencing

Bacterial 16S rRNA gene amplicons were generated using primers targeting the V3–V4 region [29], while fungal ITS amplicons targeted the ITS2 region [30]. PCR reactions (50 μL total volume) contained 1 U Phusion High-Fidelity DNA Polymerase (Biozym Scientific, Oldendorf, Germany), 5% DMSO, 0.2 mM of each primer, 200 μM dNTPs, 0.2 μL of 50 mM MgCl, and 25 ng of template DNA.

The thermal cycling profile for bacterial amplification was: 98 °C for 1 min (initial denaturation), followed by 25 cycles of 98 °C for 45 s, 60 °C for 45 s, 72 °C for 30 s, and a final extension at 72 °C for 5 min. PCR products were verified via agarose gel electrophoresis and purified using MagSi-NGSPREP Plus Magnetic Beads (Steinbrenner Laborsysteme GmbH, Wiesenbach, Germany).

Indexed libraries were prepared using the Nextera XT Index Kit (Illumina, San Diego, USA), with a 50 μL reaction consisting of 5 μL template PCR product, 2.5 μL of each index primer, 12.5 μL of 2x KAPA HiFi HotStart ReadyMix, and 2.5 μL of PCR-grade water.

Indexing PCR was performed with the following thermal cycling: 95 °C for 3 min; 8 cycles of 95 °C for 30 s, 55 °C for 30 s, 72 °C for 30 s; and a final extension at 72 °C for 5 min.

Quantification of indexed products was conducted using the Quant-iT dsDNA HS Assay Kit and Qubit fluorometer (Invitrogen GmbH, Karlsruhe, Germany). Indexed libraries were purified using MagSi-NGSPREP Plus Magnetic Beads and normalized with the Janus Automated Workstation (Perkin Elmer, Waltham, Massachusetts, USA). Sequencing was performed on the Illumina MiSeq platform using dual indexing and the MiSeq Reagent Kit v3 (600 cycles), following the manufacturer’s instructions, generating 2 × 300 bp paired-end reads.

### Sequence analysis

Bacterial and fungal datasets were analyzed independently. The DADA2 algorithm in QIIME2 was used to filter reads for quality and remove chimeric sequences [31,32]. Resulting amplicon sequence variants (ASVs) were taxonomically classified using QIIME2’s naïve Bayes classifier against the SILVA database (v138.1) and UNITE database (v9.0) [33,34].

Alpha diversity metrics (Observed Features, Faith’s Phylogenetic Diversity, and Shannon Index) and beta diversity metrics were calculated using QIIME2. Principal Coordinate Analysis (PCoA) plots were generated based on Bray–Curtis distance metrics. Differences in alpha and beta diversity between groups were assessed using the Kruskal–Wallis test and Permutational Multivariate Analysis of Variance (PERMANOVA), respectively.

Taxonomic comparisons at the family level were conducted using the Kruskal–Wallis test in R (version 4.4.1) with the ‘stats’ package [35]. ASVs unclassified at the genus level in SILVA were further analyzed using BLASTn (NCBI) with default parameters [36]. Taxonomic assignments were based on top hits with identity scores ≥97% and lowest E-values.

Functional predictions of the bacterial communities were conducted using the Tax4Fun2 package in R [37]. Tax4Fun2 converted SILVA-labeled OTUs into prokaryotic KEGG orthologs and normalized the data based on 16S rRNA gene copy numbers obtained from the NCBI genome annotations [38].

## Results

### Sequence data overview

The bacterial 16S rRNA gene and the fungal ITS region were sequenced from 12 adult Medfly gut microbial communities. Sequences that passed quality control were retained after rarefaction to 10,000 reads per sample to ensure equal sequencing depth across samples. In total, 330,957 reads were obtained for the 16S rRNA gene amplicons and 277,666 reads for the ITS region. Following paired-end merging, quality filtering, and removal of chimeric and non-target sequences, 185 distinct bacterial amplicon sequence variants (ASVs) were identified. In comparison, a slightly lower number of fungal ASVs (128) was detected. Rarefaction curves showed saturation, indicating that sequencing depth was sufficient to accurately capture the overall bacterial and fungal diversity and community composition (data not shown).

### Effect of antimicrobial treatment on the bacterial and fungal Medfly microbiome

**Fig 1:**
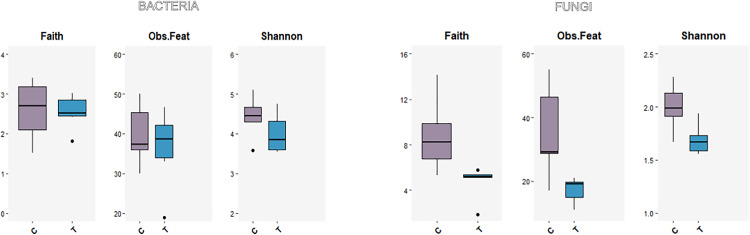
Alpha diversity indices of gut microbial communities in *C. capitata.* Boxplots show Faith’s phylogenetic diversity (Faith), observed features (Obs. Feat), and Shannon diversity index (Shannon) for bacterial (left panel) and fungal (right panel) communities in control (C) and treated (T) groups. Each box represents the distribution across three biological replicates.

Alpha diversity indices revealed notable differences between bacterial and fungal communities (Fig 1). Bacterial communities exhibited higher Shannon diversity (3.5–5.1) and Faith’s phylogenetic diversity (1.5–3.4) compared to fungal communities (Shannon: 1.6–2.3; Faith: 1.9–14.2) under both control and treated conditions (S1 Table). These results suggest that bacterial communities are inherently more diverse than their fungal counterparts.

The antimicrobial treatment led to a reduction in Shannon diversity for both bacterial and fungal communities. In bacterial samples, diversity decreased from 4.3–5.1 (controls) to 3.5–4.7 (treated), while fungal diversity dropped from 1.7–2.3 to 1.6 in most samples. This reduction was more pronounced and consistent in the fungal communities, indicating a greater sensitivity of fungi to the applied treatment.

**Fig 2:**
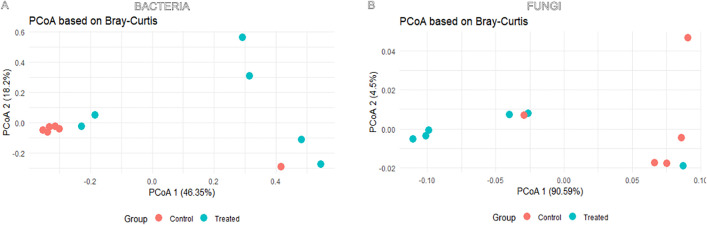
Principal Coordinates Analysis (PCoA) of gut bacterial (A) and fungal (B) communities in *C. capitata.* Plots were constructed using Bray-Curtis dissimilarity matrices based on ASV abundance data. Samples cluster by treatment group: control (red) and treated (blue).

Principal Coordinates Analysis (PCoA) based on Bray–Curtis dissimilarity revealed distinct clustering of both bacterial and fungal communities between control and treated groups. Fungal communities exhibited a more pronounced separation along the first PCoA axis, which accounted for 90% of the variance, compared to bacterial communities, where Axis 1 explained 46% of the variance (Fig 2). Statistical analysis using PERMANOVA based on UniFrac distances revealed no significant differences in community composition between males and females (p > 0.05). Therefore, samples from both sexes were pooled within control and treated groups, between which significant differences were detected with the same test for both, bacterial (p = 0.006) and fungal (p = 0.019) communities. These results demonstrate that the treatment significantly altered the composition of both bacterial and fungal microbiomes.

### Bacterial and fungal taxa of the Medfly gut

**Fig 3:**
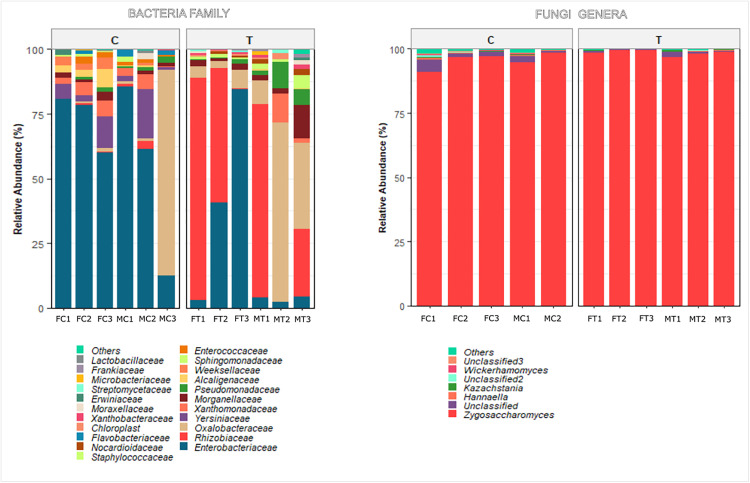
Taxonomic composition of bacterial and fungal communities in *C. capitata* gut microbiota across control (C) and treated (T) samples. **(A)** Relative abundance of bacterial families based on 16S rRNA gene sequencing. **(B)** Relative abundance of fungal genera based on ITS region sequencing. Note: In the case of bacteria families are displayed for ease of graphical representation. In the case of fungi, genera are shown given that composition was much less diverse.

The taxonomic composition of all samples is illustrated in Fig 3. Bacterial communities in both control and antibiotic-treated flies were dominated by the phylum Proteobacteria, accounting for 94% of sequences in the control group and 96% in the treated group. Although Firmicutes and Bacteroidota were not dominant phyla, both showed a decrease in relative abundance following antibiotic treatment: Firmicutes declined from 2% in the control group to 0.3% in treated samples, while Bacteroidota dropped slightly from 3% to 2%. These results suggest that antimicrobial treatment also impacted less abundant, yet ecologically relevant, bacterial members.

At the family level, Enterobacteriaceae was the most abundant in control flies (63%), but its relative abundance decreased significantly in the treated group (23%). In contrast, the relative abundance of Rhizobiaceae increased markedly from 1% in controls to 40% in treated samples. Similarly, Oxalobacteraceae increased from 13% to 20% after treatment. Notably, members of the Yersiniaceae, which represented 7% of the control group, were absent from treated samples.

**Fig 4:**
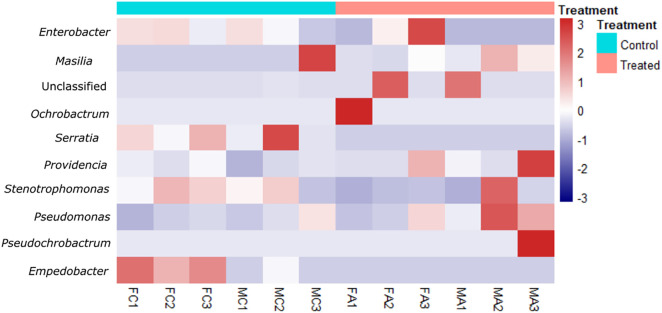
Heatmap showing the relative abundance of the ten most abundant bacterial genera in the *C. capitata* gut. Control (C) and treated (T) samples for females (F) and males (M).

A heatmap showing the most abundant genera is shown in Fig 4. Control samples were dominated by *Enterobacter* (63%), followed by *Massilia* (14%) and *Serratia* (7%). Treated samples exhibited a marked shift in dominance: *Enterobacter* decreased to 23%, while *Massilia* (21%) and an unclassified genus from the Rhizobiaceae (21.5%) became prominent. Interestingly, *Ochrobactrum* and *Pseudochrobactrum* were detected exclusively in a single treated sample. Other genera such as *Providencia*, *Stenotrophomonas*, and *Pseudomonas* were consistently present across all samples, though in lower relative abundances (~3%). Additionally, *Empedobacter*, *Enterococcus,* and *Serratia* were found only in the control group, suggesting these taxa may be sensitive to antibiotic treatment or fall below the detection threshold post-exposure.

Fungal communities were overwhelmingly dominated by the phylum Ascomycota, with an average relative abundance of 99.1% in control samples and 99.5% in antimicrobial-treated samples. Basidiomycota was detected at much lower levels, comprising 0.9% of the control group and 0.4% of the treated group. At the genus level, *Zygosaccharomyces* was overwhelmingly dominant, representing 99% of the reads across both treatment groups. This finding indicates that *Zygosaccharomyces* is a core fungal taxon in the gut microbiome of Medfly, displaying remarkable stability in response to antimicrobial perturbation. At the family level, fungal communities were almost exclusively represented (>98%) by Saccharomycetaceae, which encompasses the dominant genus *Zygosaccharomyces* already described above.

### Prevalent potential pathways in Medfly

**Fig 5:**
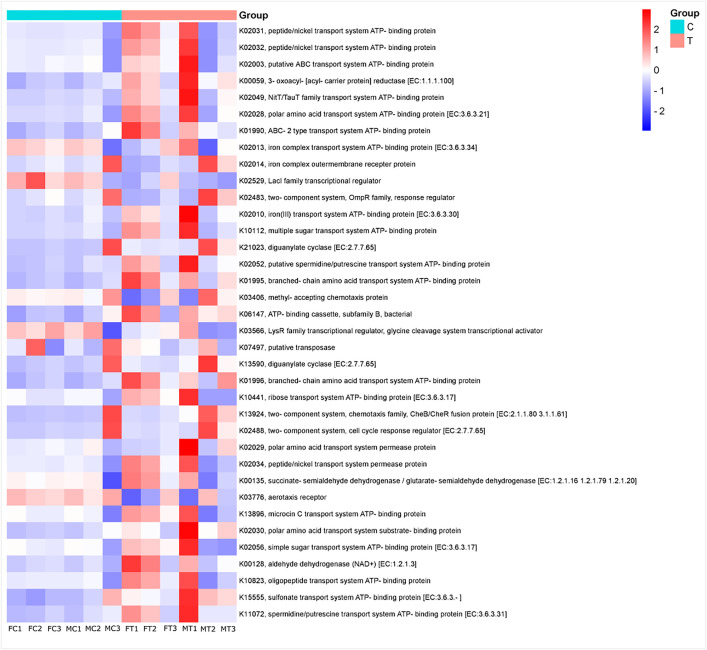
Predicted functional profiles of the *C. capitata* gut microbiota in control (C) and treated (T) samples. Heatmap displays the relative abundance of selected KEGG Orthologs (KOs) associated with metabolic functions. Predictions were performed by employing Tax4Fun (31). Functional profiles were estimated *in silico* and should be considered hypothetical. Although there were no significant differences between sexes, females (F) and males (M) are shown in the horizontal axis.

The functional predictions for all samples are shown in Fig 5. Functional profiling performed using Tax4Fun2 revealed notable shifts in microbial functional potential between control and antibiotic-treated groups. In control samples, there was a pronounced enrichment of KEGG Orthologs (KOs) associated with nutrient transport systems, including peptide/nickel transporters (K02031, K02032), ABC-type transporters (K02003, K02013), and iron and branched-chain amino acid transport proteins (K02010, K01995). These functional categories suggest that the native gut microbiota actively contribute to nutrient uptake and metal ion homeostasis, processes essential for maintaining microbial viability and mediating host–microbe interactions.

In contrast, antibiotic-treated samples exhibited an increased abundance of functions associated with stress responses and microbial adaptation. Among these were diguanylate cyclases (K21023, K13590), enzymes involved in cyclic di-GMP signaling, a key pathway regulating biofilm formation and cell cycle progression. Similarly, chemotaxis-related proteins (e.g., K03406, K13942) were more abundant in the treated group, potentially reflecting a microbial community shift toward motile or adaptive phenotypes capable of navigating altered gut environments.

These observed functional shifts suggest that antibiotic treatment not only reduces diversity but also restructures the functional potential of the gut microbiota. This reconfiguration favors traits linked to stress tolerance, environmental sensing, and survival strategies. Such changes may have important implications for the stability and resilience of the gut ecosystem, potentially affecting host physiology and susceptibility to colonization by opportunistic pathogens.

## Discussion

This study provides a comprehensive analysis of the bacterial and fungal gut microbiome of Medfly, focusing on the effects of antimicrobial treatment on these communities. Our results revealed distinct patterns in alpha and beta diversity, taxonomic composition, and the hypothetical functional potential between bacterial and fungal communities.

Proteobacteria dominated the bacterial microbiome in both control and treated groups, with Enterobacteriaceae predominating in controls. This result aligns with previous studies reporting the prevalence of Proteobacteria and Enterobacteriaceae in the Medfly gut [10,12,39–42]. However, treated samples exhibited a reduced relative abundance of Enterobacteriaceae, likely due to antibiotic exposure, and a concomitant increase in Rhizobiaceae—a family previously reported in low abundance in Australian samples [10]. Additionally, *Pseudomonas* was present in treated samples, previously linked to reduced fly longevity [42]. The observed reduction in relative abundance of Enterobacteriaceae in treated samples is likely a direct effect of antibiotic exposure, as members of this family are known to be particularly sensitive to broad-spectrum antibiotics, which disrupt cellular functions such as cell wall synthesis and protein production in Gram-negative bacteria. The reduction of Enterobacteriaceae impacts key symbiotic functions related to nutrient assimilation and host fitness. The rise of Rhizobiaceae reflects reduced competition or antibiotic resistance, while *Pseudomonas* is an opportunistic colonizer [42]. Rhizobiaceae have been found to exhibit two antibiotic resistance mechanisms: they can actively expel antibiotics and toxic metabolites through ATP propelled multi-drug efflux pumps and/or through the formation of biofilms where bacteria in biofilms resist antibiotic concentrations thanks to these extracellular matrixes [43]. Both mechanisms were inferred in our study, given that we recorded greater frequencies of ATP transport systems (e.g., ABC and binding cassette) and diguanylate cyclase in treated flies. These shifts could have implications for pest control strategies such as the Sterile Insect Technique (SIT), where microbiome composition may constitute a factor influencing insect fitness and reproduction. Both mass rearing conditions and irradiation are known to disrupt the gut microbiome [44], and larval diets often contain antimicrobials [45]. To improve sterile fly performance, bacterial probiotic adult diets have been tested, aiming to restore the microbiome after irradiation [46], though fungal strains have yet to be explored. The consistent dominance of Enterobacteriaceae reinforces its role as a core gut microbiome component in tephritid fruit flies.

This is the first study to investigate fungal diversity in the gut microbiome of *C. capitata*. Fungal communities were less diverse than bacterial ones, dominated by *Zygosaccharomyces*, a yeast genus consistently found in both control and treated samples. This aligns with our previous findings, where *Zygosaccharomyces* was the only yeast isolated from *C. capitata* in this region [24].

Yeasts, particularly *Zygosaccharomyces*, contribute significantly to protein intake and longevity in Medfly. Diets low in yeast lead to reduced protein accumulation in larvae [19], while yeast-enriched diets promote better pupal recovery and weight [47]. *Zygosaccharomyces* has been identified as a dominant yeast genus in Medfly, likely playing a crucial role in fitness and development [24]. The combined bacterial and fungal microbiome contributes to resistance against environmental stress, such as desiccation and high temperatures [24]. Yeasts also provide essential nutrients that support insect survival and fitness.

Beta diversity analyses revealed distinct clustering patterns for bacterial and fungal communities in response to antimicrobial treatment. PCoA based on Bray-Curtis dissimilarity showed a stronger separation in fungal communities, with the primary axis accounting for 90% of the variance, compared to 46% for bacterial communities (Fig 2). PERMANOVA analysis further confirmed significant differences between control and treated groups for both bacteria and fungi, indicating that antimicrobial treatment significantly altered both microbiomes.

The dominance of *Zygosaccharomyces* in Medfly may have significant ecological and physiological implications. *Z. rouxii* is known for its tolerance to osmotic pressure and acidic environments, traits that enhance resilience under fluctuating dietary conditions, such as those in decaying fruit [48]. Additionally, *Z. rouxii* produces ergosterol, which may influence reproductive fitness, as ergosterol-derived pheromones have been linked to mating behavior in fruit flies [49]. Its metabolic versatility, including fermentative metabolism, also aids energy availability and detoxification. These results suggest that the symbiotic relationship with *Zygosaccharomyces* may be a contributing factor to the fitness and adaptability of Medfly. Moreover, like the microbiota of *Drosophila melanogaster*, *Zygosaccharomyces* offers experimental accessibility, making it a promising model for studying yeast-host interactions.

KEGG pathway analysis revealed that antibiotic treatment altered bacterial metabolic potential, with changes in pathways related to biosynthesis, transport, and stress responses. The reduction in antibiotic biosynthesis pathways suggests a loss of microbial taxa associated with these functions, while the increase in ABC transporters and phosphotransferase systems indicates microbial adaptation to stress.

Together, these results highlight the complex interplay between bacterial and fungal communities in shaping the gut ecosystem of Medfly. While bacterial communities showed greater taxonomic and functional plasticity, fungal communities were more stable and dominated by *Zygosaccharomyces*. These findings underscore the need for integrated approaches to understand the microbiome’s contributions to insect physiology and ecology.

## Conclusion

This study confirms the ubiquitous presence of Enterobacteriaceae in *C. capitata* and reinforces their potential role in shaping the gut microbiome of Medflies worldwide. Antibiotic treatment affected its relative abundance, leading to a relative abundance increase of other taxa, including potential pathogens.

Additionally, this is the first study to evaluate the fungal diversity in Medfly, revealing *Zygosaccharomyces* as the dominant genus. This finding suggests a potential role of fungi in the fruit fly ecology, opening new avenues for research on host-fungal interactions and their biological implications.

From a pest management point of view, the results of this study suggest that probiotic diets aiming at increasing sterile fly performance for SIT could be more effective when including dominant bacterial and fungal groups.

## Supporting information

S1 Table Alpha diversity indices of bacterial and fungal communities associated with *C. capitata* gut samples under control (C) and antimicrobial treatment (T) conditions. The table includes the number of observed features (ASVs), Shannon diversity index, and Faith’s phylogenetic diversity (Faith) for each sample. Sample IDs are coded according to treatment (C = control, T = treatment), origin (H = head, M = midgut), and replicate number. Missing data (–) indicates unsuccessful amplification or sequencing failure.
(PDF)

S2 Table Relative abundance of bacterial genera in the gut microbiota of *C. capitata* under control (C) and antimicrobial treatment (T) conditions. Values represent the range of relative abundance observed across replicates for each genus, listed with their respective family classifications.
(PDF)
