# Supplementary material for: Dominance of Zygosaccharomyces and shifts in bacterial pathways: Effects of antimicrobials on composition and diversity of the Ceratitis capitata bacterial and fungal microbiome
Source: PLoS One. 2025 Nov 12;20(11):e0335811. doi: 10.1371/journal.pone.0335811 (PMC12611111; doi:10.1371/journal.pone.0335811)
Supplement: S1 Table — The table includes the number of observed features (ASVs), Shannon diversity index, and Faith’s phylogenetic diversity (Faith) for each sample. Sample IDs are coded according to treatment (C = control, T = treatment), origin (H = head, M = midgut), and replicate number. Missing data (–) indicates unsuccessful amplification or sequencing failure. (PDF) [file pone.0335811.s001.pdf]

S1 Table. Alpha diversity indices of bacterial and fungal communities associated with *C. capitata* gut samples under control (C) and antimicrobial treatment (T) conditions. The table includes the number of observed features (ASVs), Shannon diversity index, and Faith's phylogenetic diversity (Faith) for each sample. Sample IDs are coded according to treatment (C = control, T = treatment), origin (H = head, M = midgut), and replicate number. Missing data (–) indicate unsuccessful amplification or sequencing failure.

		**BACTERIA**			**FUNGI**		
**Treatment**	**Sample ID**	**Observed Features**	**Shannon**	**Faith**	**Observed Features**	**Shannon**	**Faith**
**C**	HC1	36	4.3	1.5	55	2.3	14.2
**C**	HC2	47.7	4.7	2.2	28.8	2.0	8.3
**C**	HC3	50	5.1	3.2	29.1	1.9	6.7
**C**	MC1	38.5	4.3	3.2	46.4	2.1	9.9
**C**	MC2	36	4.6	3.4	17	1.7	5.3
**C**	MC3	30	3.6	2.1	-	-	-
**T**	HA1	32.9	3.5	3.0	19	1.6	5.1
**T**	HA2	42.7	4.4	3.0	11	1.6	1.9
**T**	HA3	40.4	4.1	2.5	13.7	1.6	5.2
**T**	MA1	37	3.6	2.5	20	1.9	5.2
**T**	MA2	19	3.6	1.8	20.9	1.7	5.8
**T**	MA3	46.7	4.7	2.4	19.3	1.7	5.4
