# Supplementary material for: Dominance of Zygosaccharomyces and shifts in bacterial pathways: Effects of antimicrobials on composition and diversity of the Ceratitis capitata bacterial and fungal microbiome
Source: PLoS One. 2025 Nov 12;20(11):e0335811. doi: 10.1371/journal.pone.0335811 (PMC12611111; doi:10.1371/journal.pone.0335811)
Supplement: S2 Table — Values represent the range of relative abundance observed across replicates for each genus, listed with their respective family classifications. (PDF) [file pone.0335811.s002.pdf]

S2 Table. Relative abundance of bacterial genera in the gut microbiota of *C. capitata* under control (C) and antimicrobial treatment (T) conditions. Values represent the range of relative abundance observed across replicates for each genus, listed with their respective family classifications.

**Genus (Family)**	**Relative abundance**	
	C	T
Enterobacter (Enterobacteriaceae)	12.4 - 85.5	2.4 - 84.3
Massilia (Oxalobacteraceae)	0.8 - 79.5	2.6 - 79.2
**Unclassified** (Rhizobiaceae)	0.5 - 2.9	1.6 - 74.7
Ochrobactrum (Rhizobiaceae)	0	0 - 85.9
Serratia (Yersiniaceae)	1 - 19.1	0
Providencia (Morganellaceae)	1.4 - 3.4	0.8 - 13.0
Stenotrophomonas (Xanthomonadaceae)	0.4 - 4.9	0.2 - 1.4
Pseudomonas (Pseudomonadaceae)	0.7 - 1.5	0.5 - 6.3
Pseudochrobactrum (Rhizobiaceae)	0	24.4
Empedobacter (Weeksellaceae)	0.8 - 4.4	0
